# Taxonomy and Molecular Phylogeny of Two New Species of Prostomatean Ciliates With Establishment of *Foissnerophrys* gen. n. (Alveolata, Ciliophora)

**DOI:** 10.3389/fmicb.2021.686929

**Published:** 2021-06-18

**Authors:** Limin Jiang, Wenbao Zhuang, Hamed A. El-Serehy, Saleh A. Al-Farraj, Alan Warren, Xiaozhong Hu

**Affiliations:** ^1^College of Fisheries and Key Laboratory of Mariculture, Ministry of Education, Ocean University of China, Qingdao, China; ^2^Institute of Evolution and Marine Biodiversity, Ocean University of China, Qingdao, China; ^3^Department of Zoology, College of Science, King Saud University, Riyadh, Saudi Arabia; ^4^Department of Life Sciences, Natural History Museum, London, United Kingdom

**Keywords:** biodiversity, ciliated protozoa, intracellular prokaryote, molecular systematics, new taxa, SSU rRNA gene

## Abstract

Prostomatean ciliates play important roles in the flow of material and energy in aquatic microbial food webs, and thus have attracted wide attention for over a century. Their taxonomy and systematics are, however, still poorly understood because of their relatively few taxonomically informative morphological characters. In this study, two new prostomateans, *Lagynus binucleatus* sp. n. and *Foissnerophrys alveolata* gen. n., sp. n., collected from a freshwater pool and the intertidal zone of a sandy beach, respectively, in Qingdao, China, are investigated using living observation, protargol staining, and SSU rRNA gene sequencing methods. The genus *Lagynus* is redefined, and the new species *L. binucleatus* sp. n. is established based on significant morphological differences with similar forms. Furthermore, a new genus, *Foissnerophrys* gen. n., is established based on a combination of morphological and molecular data with *F. alveaolata* sp. n. the type species by monotypy. The identities of intracellular prokaryotes of these two new species are discussed based on fluorescence *in situ* hybridization (FISH) data and newly obtained 16S rRNA gene sequences.

## Introduction

The class Prostomatea Schewiakoff, 1896 is one of the smallest in the phylum Ciliophora Doflein, 1901 in terms of species and genus richness (Lynn, 2008). Nevertheless, its members are commonly found in freshwater, marine, and soil habitats worldwide (Kahl, 1930; Carey, 1992; Foissner et al., 1994, 1999; Song et al., 2009; Chen et al., 2010, 2012; Hu et al., 2019; Sikder and Xu, 2020). As abundant components of aquatic ecosystems, prostomateans play several important ecological roles utilizing a wide range of bacteria and microalgae as food (Madoni et al., 1990; Epstein and Shiaris, 1992; Šimek et al., 1996; Weisse and Montagnes, 1998; Nakamura and Hirata, 2006; Lynn, 2008). For example, the marine genus *Tiarina* Bergh, 1881 has been reported to control the growth of algae that cause red tides (Jeong et al., 2002), while the ectoparasite *Cryptocaryon irritans* Brown, 1951 can cause skin diseases in marine fishes (Dickerson and Dawe, 2006). Symbiotic partnerships between ciliate hosts and intracellular bacteria or microalgae have also been recorded in this group. For example, symbiotic bacteria have been discovered in *Urotricha ovata* Kahl, 1926 (de Puytorac and Grain, 1972), and the microalga *Symbiodinium* has been found in a calcifying *Tiarina* (Mordret et al., 2016). However, species identification and evolutionary relationships of prostomateans remain difficult due to the lack of ciliature information and molecular data.

Corliss (1979) assigned prostomateans to a single order in the subclass Gymnostomatia Corliss, 1974 (class Kinetofragminophora de Puytorac et al., 1974). Based on ultrastructural data and the tube-like cytopharynx (“rhabdos”), Small and Lynn (1985) united the classes Prostomatea Schewiakoff, 1896 and Litostomatea Small and Lynn, 1981 into the subphylum Rhabdophora Small, 1976. In the classification system proposed by Lynn (2008), the class Prostomatea contains two orders: Prostomatida Schewiakoff, 1896 and Prorodontida Corliss, 1974.

The order Prostomatida is characterized by having a truly apical oral region, perioral kineties that form obvious paratenes, and the absence of both a brosse and toxicysts (Lynn, 2008). Prostomatida contain only two families, namely, Metacystidae Kahl, 1926 and Apsiktratidae Foissner et al., 1994. Metacystids can be recognized by their bipolar somatic kineties, conspicuous transverse perioral kineties surrounding the cytostome and, in some species, the presence of a lorica (Small and Lynn, 1985). Based on body shape and the presence or absence of a monokinetidal oral ring, Metacystidae are divided into three genera, namely, *Metacystis* Cohn, 1866, *Vasicola* Tatem, 1869, and *Pelatractus* Kahl, 1930 (Kahl, 1930; Small and Lynn, 1985). Despite a long research history, comparatively little information is available for metacystids and only a few species, all belonging to the genus *Metacystis*, have been reported in detail (Kahl, 1930; Small and Lynn, 1985; Song and Wilbert, 2002; Aladro-Lubel and Martínez-Murillo, 2003; Arregui et al., 2010; Zhang et al., 2015).

The order Prorodontida is characterized by the oral region that is located apically or slightly subapically and by the presence of both a brosse and toxicysts (Lynn, 2008). It contains nine families including Lagynidae Sola et al., 1990, the type genus of which is *Lagynus* Quennerstedt, 1867. *Lagynus* is easily distinguished from most other prostomateans by having a bottle-shaped or fusiform body and an annular neck. Quennerstedt (1867) established *Lagynus* to accommodate those species of *Lacrymaria* Bory de Saint Vincent, 1824, that are only slightly contractile. *Lagynus elegans* (basionym: *Lacrymaria elegans* Engelmann, 1862) was designated as the type species. Kahl (1930), however, did not accept this classification, so he transferred all species of *Lagynus* to the genera *Lacrymaria* Bory de Saint Vincent, 1824, *Enchelys* Müller, 1773, *Enchelyodon* Claparède and Lachmann, 1859, and *Trachelocerca* Ehrenberg, 1840. Corliss (1979) considered *Lagynus* to be a *nomen nudum*, so this genus was not accepted in his systematic revision of ciliates. Following further investigations, Foissner (1983) and Sola et al. (1990) confirmed that *Lagynus* differs significantly from *Lacrymaria* in having a crown of nematodesmata surrounding the oral region and a short brosse that is not attached to the somatic kineties and hence differs from the dorsal brush in Haptorida Corliss, 1974. Consequently, Sola et al. (1990) established the new family Lagynidae for *Lagynus*. Foissner et al. (1995) subsequently confirmed the taxonomic status of this genus. Nevertheless, of the three nominal *Lagynus* species, only *L. elegans* has been described in detail. Moreover, the lack of molecular data has hampered knowledge and understanding of the systematics of *Lagynus*. This genus should be, therefore, redefined based on new information.

In the present work, two new species, one prostomatid and one prorodontid are described, and their molecular phylogenies based on SSU rRNA gene sequence data are analyzed. In addition, their intracellular prokaryotes are investigated by fluorescence *in situ* hybridization (FISH) and 16S rRNA gene sequencing in order to better understand the ecological function of these ciliates in aquatic ecosystems.

## Materials and Methods

### Sample Collection and Morphological Methods

**FIGURE 1:**
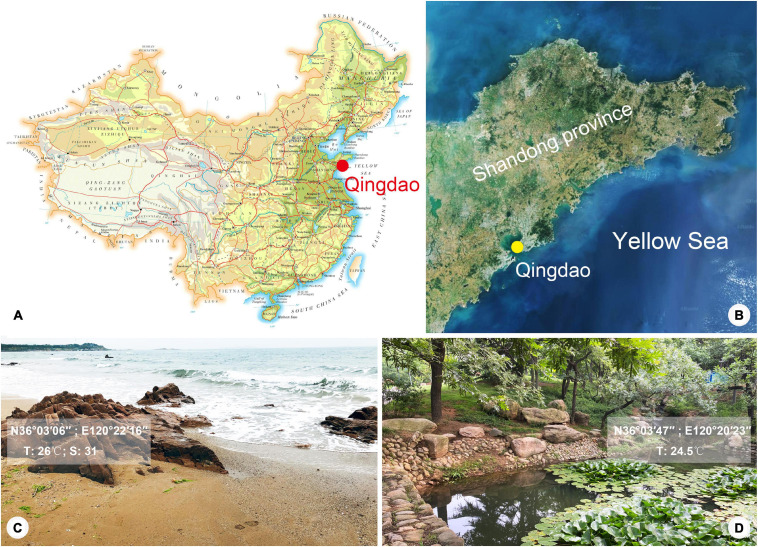
Map and satellite image showing location of Qingdao and photographs showing sampling sites. **(A)** Map of China, showing location of Qingdao. **(B)** Satellite image of Shandong Province, showing location of Qingdao. **(C,D)** Photographs of sampling sites in Qingdao where *Foissnerophrys alveolata* gen. n., sp. n. **(C)** and *Lagynus binucleatus* sp. n. **(D)** were collected.

*Lagynus binucleatus* sp. n. was collected from a freshwater pond in the Zhongshan Park, Qingdao, China (36°03′47″N, 120°20′23″E), on October 17, 2019, when the water temperature was 24.5°C. *Foissnerophrys alveolata* gen. n., sp. n. was collected from the intertidal zone of a sandy beach at the Taipingjiao Park, Qingdao, China (36°03′06″N, 120°22′16″E), on August 26, 2019, when the water temperature was 26°C, and the salinity was 31‰ (Figure 1). Water samples with sediment or sand were collected using bottle caps after gently stirring the water. In the case of the freshwater pond, sediment samples were taken from the surface layer of the pond-bed using a Pasteur pipette and then diluted with untreated habitat water.

After collection, samples were transferred to Petri dishes. Living cells were isolated from the cultures with a micropipette and observed at 100–1000× magnification using bright field and differential interference contrast microscopy. The protargol staining method described by Wilbert (1975) was used to reveal the ciliature and nuclear apparatus. The protargol powder was made according to Pan et al. (2013). Morphometric measurements and counts were performed at a magnification of 1,000×. Drawings of living cells were produced using freehand sketches and photomicrographs, while drawings of silver-stained specimens were produced with the help of a drawing device. Terminology and systematics are mainly according to Foissner et al. (1995).

### Terminology

Brosse: distinctive field of clavate cilia arising from three or more specialized kineties or kinetal segments, often oriented obliquely to the body axis; characteristically found in prorodontine prostomes.

Paratene: condition or presence of recognizable repeating kinetid patterns that are oriented orthogonal to the longitudinal axis of the ciliate’s body, thus parallel to the equator or eventual fission furrow; paratenes superficially give the impression that the organism’s kineties run circumferentially rather than longitudinally in the part of the body affected.

Cervical kineties (CeK): paratenes composed of comparatively loosely arranged kinetosomes in the “neck” region, i.e., between perioral kineties and ordinary somatic kineties.

Perioral kineties (PK): paratenes composed of closely arranged kinetesomes between circumoral kinety and cervical kineties.

### DNA Extraction, PCR Amplification, and Sequencing

Single cells were isolated under the dissecting microscope and washed in filtered water from the original sample (Bai et al., 2020). Extraction of genomic DNA from a single cell was performed using DNeasy Blood and Tissue Kit (QIAGEN, Hilden, Germany) following the manufacturer’s instructions (Wu et al., 2020). The genomic DNA was stored at −80°C prior to further processing. Q5 Hot Start high fidelity DNA polymerase (NEB, Ipswich, MA, USA) was used to amplify the SSU rRNA gene using primers Euk-A (5′-AAYCTGGTTGATYYTGCCAG-3′) and Euk-B (5′-CYGCAGGTTCACCTACRG-3′), resulting in a near-complete SSU rDNA fragment (Medlin et al., 1988). Cycling parameters of PCR amplification were as follows: one cycle of initial denaturation at 98°C for 30 s, followed by 18 cycles of amplification (98°C, 10 s; 69°–51°C touchdown, 30 s; 72°C, 1 min), and another 18 cycles (98°C, 10 s; 51°C, 30 s; 72°C, 1 min), with a final extension of 72°C for 5 min (Jiang et al., 2021). The PCR products were sequenced directly in both directions by four reactions (18SF, 18SR, 900F, and 900R) by TSINGKE (Qingdao, China). Contigs were assembled using Seqman (DNAStar).

For intracellular bacteria, the 16S rRNA gene was amplified using primers Bacteria-16SF (AGAGTTTGATCATGGCTCAG) and Bacteria-16SR (TAGGGTTACCTTGTTACGACTT) according to Medlin et al. (1988) and Miller et al. (2014). For archaea, the 16S rRNA gene was amplified using Pro-341F (CCTACGGGNBGCASCAG) and Pro-805R (GACTACNVGGGTATCTAATCC) (Takahashi et al., 2014). The parameters of PCR amplification were the same as those for 18S. The PCR products were purified by EasyPure PCR Purification Kit (TransGen Biotech, China) and then cloned by pClone007 Blunt Simple Vector Kit (Tsingke, China). Transformed cells were grown overnight at 37°C in LB media with 0.05 mg/ml ampicillin. Two individuals of ciliates were examined, and for each individual, eight clones were selected randomly and sequenced directly in both directions by two reactions (Bacteria-16SF, Bacteria-16SR) by TSINGKE (Qingdao, China). To ensure the credibility of the results, only the samples with high similarity (99.70% sequence similarity threshold) to known sequences from NCBI were considered in our analyses. The 16S rRNA gene sequences obtained in this study are deposited in the GenBank database under accession numbers MW979568–MW979575.

### Phylogenetic Analyses

In addition to the newly obtained SSU rRNA gene sequences of *Lagynus binucleatus* sp. n. and *Foissnerophrys alveolata* gen. n., sp. n., another 110 sequences downloaded from the GenBank were used in the phylogenetic analyses (for accession numbers, see Figure 6). The compressed subtree contains 12 populations of *Coleps viridis* (accession numbers: MT253672–MT253675, MT253678–MT253685). The oligohymenophoreans *Anoplophrya marylandensis* (AY547546), *Frontonia vernalis* (U97110), *Paramecium calkinsi* (AF100301), and *Paratetrahymena parawassi* (FJ876969) were chosen as outgroup taxa. All sequences were aligned using the MUSCLE program at the European Bioinformatics Institute (available at http://www.ebi.ac.uk/Tools/msa/muscle/). The primers on both ends of the resulting alignment were trimmed using the BioEdit 7.2.0.5 program (Hall, 1999; Zhang et al., 2020). The final alignment used for the phylogenetic analyses had 1,857 nucleotide positions.

Maximum likelihood (ML) analysis was carried out using RAxML-HPC2 v.8.2.10 on XSEDE on the CIPRES Science Gateway with GTR + I + G as the optimal model (Stamatakis, 2014). Bayesian inference (BI) analysis was performed with MrBayes v.3.2.6 on XSEDE (Ronquist et al., 2012) on the CIPRES Science Gateway, using the GTR + I + G model as selected by MrModeltest v.2.2 according to the Akaike Information Criterion (AIC) for BI (Nylander, 2004). Markov chain Monte Carlo (MCMC) simulations were then run with two sets of four chains for 10,000,000 generations at a sampling frequency of 100 and a burn-in of 25,000 trees (25%). All remaining trees were used to calculate the posterior probabilities using a majority rule consensus. MEGA v.6.0 (Kumar et al., 2016) analyses were used to visualize the tree topologies.

### Whole-Cell Fluorescence *in situ* Hybridization (FISH) and DAPI Staining

The procedure used for FISH basically followed those of Fried et al. (2002); Gong et al. (2016), and Zhao et al. (2020). The probes for bacteria were a mixture of universal prokaryote probes, Eub338, I, II, and III (Amann et al., 1995; Daims et al., 1999), and the probe for archaea was Arc915 (Raskin et al., 1994). Cells were washed in sterile water and then fixed with filtered Bouin’s solution (50% final concentration) on glass microscope slides (MATSUNAMI, FRR-04). Slides with ciliates were placed in the dark for at least 24 h and then washed with sterile water three times. Cells were dehydrated in a graded series of 30, 50, 80, and 100% ethanol (5 min each step). The cells were covered with hybridization buffer using the preparation method described by Zhao et al. (2020) and incubated at 46°C for 2 h. After hybridization, the cells were eluted at 48°C for 30 min with wash buffer and rinsed with deionized water. Finally, DAPI (50 ng/ml) was added onto the slides to stain DNA. All steps were performed in the dark. The slides were observed under a confocal laser scanning microscope at excitation wavelengths of 450–490, 546, and 365 nm for the Eub, Arc, and DAPI signals, respectively.

## Results

### ZooBank Registration

Present work: LSIDurn:lsid:zoobank.org:pub:87ABF569-9C0B-4873-9DFA-BA26FD5A45C6.
*Lagynus binucleatus* sp. n.: LSIDurn:lsid:zoobank.org:act:FBB 14E3C-0BAF-4BD8-B634-41B5425BFDBD.
*Foissnerophrys* gen. n.: LSIDurn:lsid:zoobank.org:act:EAD3B6 72-72F2-4001-BFFC-B64C77193274.
*Foissnerophrys alveolata* gen. n., sp. n.: LSIDurn:lsid: zoobank.org:act:85D2325F-F757-499F-86F1-832B5C6B4ED4.

### Morphological Descriptions

Class: Prostomatea Schewiakoff, 1896
Order: Prorodontida Corliss, 1974
Family: Lagynidae Sola et al., 1990
Genus: *Lagynus* Quennerstedt, 1867

#### Improved Diagnosis

The body was bottle-shaped or spindle-shaped, rounded in cross-section; cytostome apical; neck region encircled by furrows and slightly contractile; macronuclear nodule(s) ovoidal; circumoral kinety composed of dikinetids; three or four brosse kineties; perioral kineties present; and caudal cilium absent.

**FIGURE 2:**
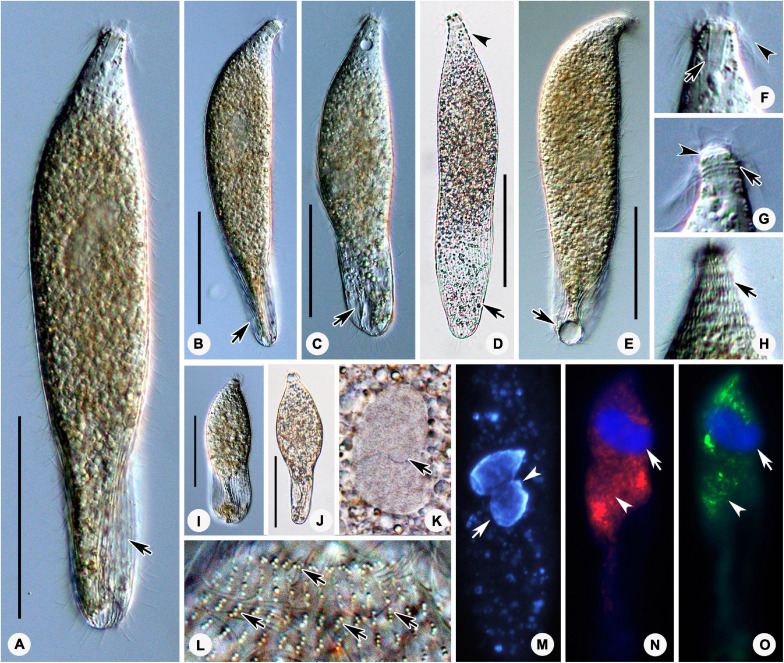
Photomicrographs of *Lagynus binucleatus* sp. n. *in vivo* **(A–L)**, after DAPI staining **(M)**, and after fluorescence *in situ* hybridization (FISH) **(N–O)**. **(A)** Lateral view of a representative individual, showing the flat posterior end of the body (arrow). **(B–D)** Shape variants, the flat posterior end of the body at different angles relative to the main body axis (arrows) and furrows in the neck region (arrowhead). **(E)** The contractile vacuole (arrow). **(F)** Side view of anterior portion of the cell, showing the nematodesmata (arrow) and perioral cilia (arrowhead). **(G)** Side view of the anterior portion of the cell, showing annular furrows (arrow) and oral bulge (arrowhead). **(H)** Side view of anterior portion of the cell, showing the furrows around the neck. **(I,J)** Showing a smaller individual. **(K)** The macronuclear nodules and micronucleus (arrow). **(L)** The cortical granules (arrows). **(M)** The macronuclear nodules (arrow) and micronucleus (arrowhead). **(N,O)** The macronuclear nodules (arrows), bacteria **(N)**, and archaea **(O)** scattered in the cytoplasm (arrowheads). Scale bars: 70 μm **(A–E)**, 90 μm **(I,J)**.

**FIGURE 3:**
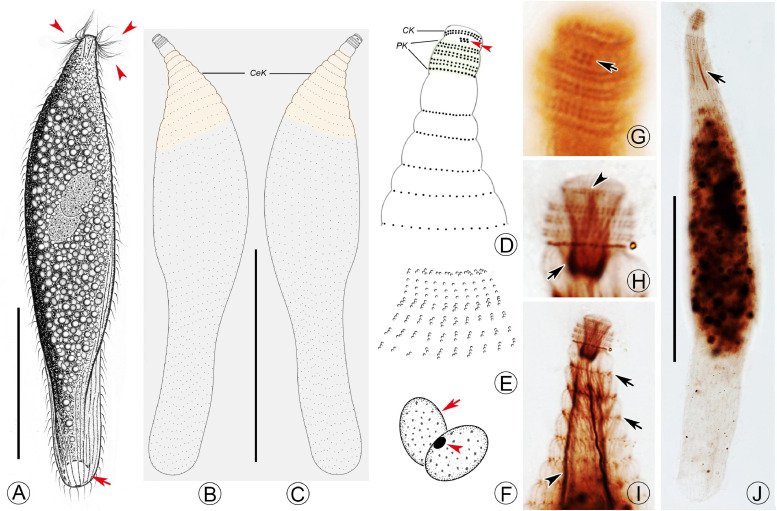
Drawings **(A–F)** and photomicrographs **(G–J)** of *Lagynus binucleatus* sp. n. *in vivo* **(A)** and after protargol staining **(B–J)**. **(A)** Lateral view of a representative individual, showing general appearance of body, the contractile vacuole (arrow), and the perioral cilia (arrowheads). **(B,C)** Side views of the holotype specimen, showing ciliary pattern of the cell and cervical kineties (CeK). **(D)** Ciliary pattern of the anterior portion of the cell, showing the circumoral kinety (CK), brosse (double arrowhead), and perioral kineties (PK). **(E)** The distribution of cortical granules. **(F)** Showing the macronuclear nodules (arrow) and micronucleus (arrowhead). **(G)** Side view of the anterior portion of the cell, showing the brosse (arrow). **(H)** Side view of the anterior portion of the cell, showing the circumoral kinety (arrowhead) and oral basket (arrow). **(I)** Side view of the anterior portion of the cell, showing the cervical kineties (arrows) and nematodesmata (arrowhead). **(J)** Lateral view, showing ciliary pattern and nematodesmata (arrow). CK, circumoral kinety; CeK, cervical kineties; PK, perioral kineties. Scale bars: 70 μm **(A)**, 120 μm **(B,C)**, 80 μm **(J)**.

*Lagynus binucleatus* sp. n.
(Figures 2A–O, 3A–J and Table 1)

**TABLE 1:** Morphometric data on *Lagynus binucleatus* sp. n. and *Foissnerophrys alveolata* gen. n., sp. n. after protargol staining. Measurements in μm.

Character	Min	Max	M	Mean	SD	CV	N
Body, length	153	310	241	237.0	38.4	16.2	30
	86	164	106	112.9	21.9	19.4	30
Body, width	21	54	39	41.1	13.3	32.4	30
	28	59	42	43.1	9.5	22.1	30
Ratio of body length to body width	5	9	6	6.3	1.9	30.7	30
	2	4	3	2.7	0.4	15.8	30
Ma, number	2	2	2	2.0	0	0	30
	1	1	1	1.0	0	0	30
Ma, length	15	25	21	20.4	2.9	14.2	30
	16	26	19	19.4	2.8	14.3	30
Ma, width	8	22	11	11.8	2.7	23.1	30
	10	23	15	15.1	2.8	18.8	30
Ratio of Ma length to Ma width	1	2	2	1.8	0.3	16.5	30
	1	2	1	1.3	0.1	12.7	30
SK, number	28	38	33	33.1	2.7	8.0	30
	41	47	44	44.0	2.1	4.7	30
Circumoral kineties, number	1	1	1	1.0	0	0	30
	1	1	1	1.0	0	0	30
Perioral kineties, number	4	4	4	4.0	0	0	30
	5	5	5	5.0	0	0	30
Cervical kineties, number	8	14	11	11.1	1.5	13.7	30
	2	3	2	2.3	0.5	20.3	30
Brosse rows, number	3	3	3	3.0	0	0	30
	–	–	–	–		–	–
OB, length	8	11	9	9.5	0.7	7.7	30
	–	–	–	–	–	–	–
OB, width	3	6	5	4.6	0.6	13.8	30
	–	–	–	–		–	–
Ratio of OB length to OB width	2	3	2	2.1	0.2	10.4	30
	–	–	–	–		–	–
Nematodesmata, length	21	38	30	30.3	4.4	14.5	30
	–	–	–	–		–	–

*CV, coefficient of variation (%); M, median; Ma, macronuclear nodules; Max, maximum; Min, minimum; n, number of specimens examined; OB, oral basket; SD, standard deviation; SK, somatic kineties; –, no data available.*

#### Diagnosis

Cell size was 165–340 × 20–60 μm *in vivo*; with two macronuclear nodules, one micronucleus; contractile vacuole terminal; brosse consists of three pairs of kinetids; four perioral kineties, anterior three double-rowed, posterior one single-rowed; 8–14 cervical kineties; and 28–38 somatic kineties.

#### Etymology

The species-group name *binucleatus* (Latin adjective; having two nuclei) is a composite of the Latin numeral *bi*- (two) and Latin adjective *nucleatus* (kernel-like), and refers to the two macronuclear nodules, a distinguishing feature of this species.

#### Type Locality and Habitat

The locality and habitat are a freshwater pond in the Zhongshan Park, Qingdao (36°03′47″N, 120°20′23″E), and the water temperature was about 24.5°C.

#### Deposition of Type Slide

One protargol slide containing the holotype specimen (registration number: JLM2019101701-1), and two protargol slides (registration numbers: JLM2019101701-2, 2019101701-3) containing several paratype specimens, were deposited in the Laboratory of Protozoology, Ocean University of China.

#### Description

The body is about 165–340 × 20–60 μm *in vivo*; the ratio of length to width is about 5–9:1 (Figures 2A–E,I,J, 3A and Table 1). The cell is generally fusiform and progressively narrows from middle toward the anterior and posterior ends both *in vivo* and after protargol staining; flexible, slightly contractile especially in the neck region, and both contraction and extension occur slowly (Figures 2A–J, 3A). The main anterior part of the cell is cylindrical and wider than the rest of the body; the posterior 25–35% of the body is flattened, transparent with almost no inclusions, and with distinct striations on the surface (Figures 2A–J). There are three distinct annular striations between the oral bulge and neck (Figure 2G). Inconspicuous step-like furrows are found around the neck (Figure 2D), and there are densely arranged longitudinal striations between furrows (Figure 2H). The pellicle is conspicuously notched with longitudinal ridges (Figures 2A–C). The ectoplasm is transparent, with numerous spherical, colorless cortical granules (about 0.5 μm in diameter) densely arranged in longitudinal rows and separated into several groups, and each group with one to five granules (Figures 2L, 3E). The cytoplasm is colorless, packed with brown granules and small spheres (2–6 μm in diameter) in the mid-region of the body rendering the cell gray at low magnifications (Figures 2D,J). There are invariably two ellipsoidal macronuclear nodules, closely apposed in the mid-region of cell, and each nodule is about 14–23 × 8–14 μm *in vivo*, 15–25 × 8–22 μm after protargol staining (Figures 2K,M, 3F and Table 1). There is a single ellipsoidal micronucleus, approximately 5–6 × 3–4 μm *in vivo*, closely associated with, sometimes located between, macronuclear nodules (Figures 2K,M, 3F). The single contractile vacuole is terminally positioned, about 14–21 μm in diameter when fully expanded (Figure 2E). Locomotion is by swimming moderately fast in the upper layer of water, either with the anterior end swinging from side to side or along a helical trajectory by rotating about the main body axis.

The somatic cilia are 10- to 13-μm long, densely arranged. In total, there are 28–38 somatic kineties, each composed of densely packed monokinetids that are arranged regularly in the anterior body half and irregularly in the posterior half (Figures 3B,C,J and Table 1). The brosse consists of three pairs of kinetids (Figures 3D,G and Table 1). There are 8–14 cervical kineties, distributed along furrows that encircle the neck region, composed of monokinetids (Figures 3B,C,I, and Table 1).

The oral bulge is rather conspicuous because it is distinctly projecting from the body proper, especially in protargol-stained specimens (Figures 2G, 3G,H); the cytostome is situated in the center of the oral bulge. The oral basket is well-developed (about 10 μm × 5 μm) with clearly visible nematodesmata, each about 10- to 13-μm long (Figures 2F, 3H–J and Table 1). There is one circumoral kinety composed of dikinetids, with each pair of kinetosomes vertically aligned (Figures 3D,H and Table 1). There are four perioral kineties, three anterior rows composed of dikinetids, with the posterior-most row composed of monokinetids; the perioral cilia, which emerge from the anterior end of the cell are conspicuous and approximately 15- to 18-μm long (Figures 2F, 3D,G,H, and Table 1).

Class: Prostomatea Schewiakoff, 1896
Order: Prostomatida Schewiakoff, 1896
*Foissnerophrys* gen. n.

#### Diagnosis

The body is spindle-shaped to cylindrical; the circumoral kinety is composed of dikinetids; perioral kineties are present; brosse, contractile vacuole, caudal cilium, and lorica are absent.

#### Dedication

This genus is dedicated to Prof. Dr. Wilhelm Foissner (Salzburg University) in acknowledgment of his achievements in the field of ciliate taxonomy. The name is a composite of the surname Foissner and the Greek noun *ophrys* (eyebrow∼cilia∼ciliate) and has a feminine gender.

#### Type Species

*Foissnerophrys alveolata* sp. n.

#### Species Assigned

*Foissnerophrys alveolata* sp. n.

**FIGURE 4:**
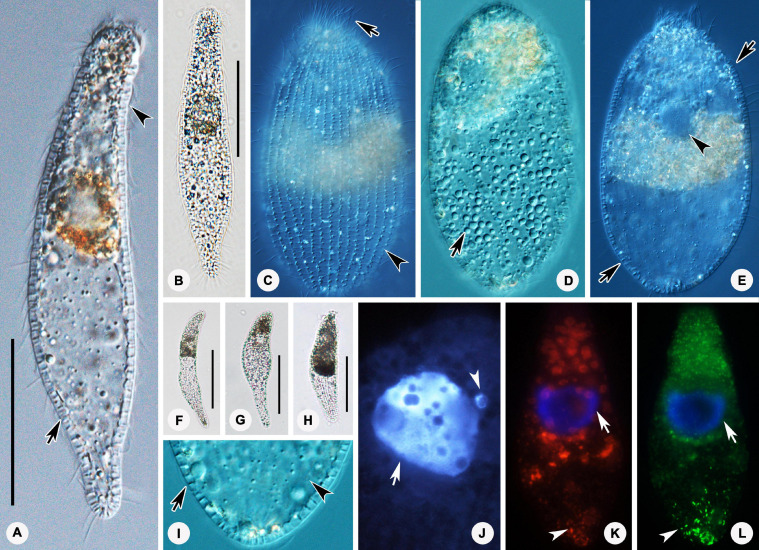
Photomicrographs of *Foissnerophrys alveolata* gen. n., sp. n. *in vivo* **(A–I)**, after DAPI staining **(J)** and after fluorescence *in situ* hybridization (FISH) **(K,L)**. **(A,B)** Lateral view of a representative individual showing the cortical granules (arrow) and furrows in ectoplasm (arrowhead). **(C)** The pattern of silverline system, perioral cilia (arrow), and cortical granules (arrowhead). **(D)** The spherical inclusions in the cell. **(E)** The macronucleus (arrowhead) and cortical granules (arrows). **(F–H)** Shape variants. **(I)** The flat posterior end of the body (arrowhead) and cortical granules (arrow). **(J)** The macronucleus (arrow) and micronucleus (arrowhead). **(K,L)** The macronucleus (arrows), bacteria **(K)**, and archaea **(L)** scattered in the cytoplasm (arrowheads). Scale bars: 40 μm **(A,B)**, 60 μm **(F–H)**.

**FIGURE 5:**
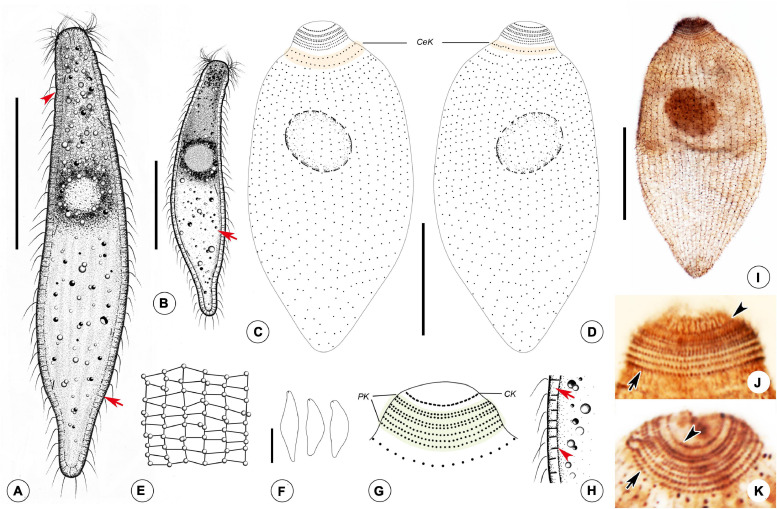
Drawings **(A–H)** and photomicrographs **(I–K)** of *Foissnerophrys alveolata* gen. n., sp. n. *in vivo* **(A,B,E,F,H)** and after protargol staining **(C,D,G,I,K)**. **(A)** Lateral view of a representative individual, showing general appearance of body, cortical granules (arrow), and furrows in ectoplasm (arrowhead). **(B)** Different body shape, showing the flat posterior end of the body (arrow). **(C,D)** Side views of the holotype specimen, showing ciliary pattern of the cell and cervical kineties (CeK). **(E)** The distribution of cortical granules and pattern of silverline system. **(F)** Lateral view of a specimen showing its contractility. **(G)** Ciliary pattern of the anterior portion of the cell showing the circumoral kinety (CK) and perioral kineties (PK). **(H)** Portion of cortex, showing the hyaline ectoplasm (arrow) and cortical granules (arrowhead). **(I)** Lateral view of holotype specimen showing ciliary pattern and macronucleus. **(J,K)** Side view of anterior portion of the cell showing the circumoral kinety (arrowheads) and perioral kineties (arrows). CeK, cervical kineties; CK, circumoral kinety; PK, perioral kineties. Scale bars: 40 μm **(A–D,I)**, 60 μm **(F)**.

*Foissnerophrys alveolata* gen. n., sp. n.
(Figures 4A–L, 5A–K, and Table 1)

#### Diagnosis

The cell size is 75–150 × 20–45 μm *in vivo*; the body is elongate with a conspicuous alveolar layer covering the surface; with single macronucleus and single micronucleus; five perioral kineties, two anterior double rowed, three posterior single rowed; two or three cervical kineties; and 41–47 somatic kineties.

#### Etymology

The species-group name *alveolata* (Latin adjective) is a composite of the Latin noun *alveolus* (vesicle) and the suffix ∼*ata* (having something) and refers to the alveolar layer, a main distinguishing feature of this species.

#### Type Locality and Habitat

The locality and habitat are the intertidal zone of a sandy beach at the Taipingjiao Park, Qingdao (36°03′06″N, 120°22′16″E), China. The salinity is 31‰, water temperature is about 26°C.

#### Deposition of Type Slide

One protargol slide containing the holotype specimen (registration number: JLM2019082602-1), and two protargol slides (registration numbers: JLM2019082602-2, 2019082602-3) containing several paratype specimens, were deposited in the Laboratory of Protozoology, Ocean University of China.

#### Description

The cell size is about 75–150 × 20–45 μm *in vivo* when fully extended, with a length-width ratio of about 3–5:1 (Figures 4A,B, 5A,B and Table 1). The cell is flexible and contractile (Figure 5F). The body shape is highly variable among different individuals, usually spindle shaped, progressively narrowed from middle to both ends, with a rounded head (Figures 4A,B,F–H, 5A,B). Distinct furrows appear in the neck region when the body contracts (Figures 4A, 5A). Single ellipsoidal macronucleus are located in the mid-body region, 12–14 μm across *in vivo*, 16–26 × 10–23 μm after protargol staining (Figures 4E,J–L and Table 1). Single globular micronucleus adjacent to the macronucleus, about 2 μm in diameter, are observable after DAPI staining (Figure 4J). No contractile vacuole is observed. Cortical granules are colorless, rod-shaped, about 2.0–2.5 × 0.7–0.8 μm, equally spaced and distributed regularly in longitudinal rows, oriented orthogonal to the cell membrane (Figures 4A,C,I, 5E,H). A pattern of the silverline system is easily recognized *in vivo*, consisting of one polygon between each kinety (Figures 4C, 5E). The ectoplasm is hyaline and flexible, forming a clear margin around the endoplasm, distinctly furrowed in the neck region (Figures 4A,I). The cytoplasm is colorless, but the anterior portion of the body is usually opaque and grayish due to the presence of numerous highly refractive inclusions and cytoplasmic granules, ellipsoidal or globular, ca. 1–3 μm in diameter (Figures 4D,E); the remainder of the cell is nearly transparent, with sparsely scattered granules (Figures 4E,I, 5A,B). Locomotion is by swimming slowly with the anterior end swinging from side to side.

The somatic cilia are about 6–8 μm long *in vivo*. In total, there are 41–47 somatic kineties, mainly consisting of monokinetids and extending almost through the entire body length (Figures 5C,D,I and Table 1). In the anterior half of the body, somatic kinetosomes are arranged in regular kineties, but in the posterior half of the body, kinetosomes are scattered in an irregular pattern (Figures 5C,D,I). Two or three rather indistinct cervical kineties encircle the neck region between the perioral kineties and the anterior end of the somatic kineties (Figures 5C,D and Table 1).

The cytostome is apical, and the oral bulge is indistinct *in vivo* but noticeably convex after protargol staining (Figure 5I). The oral basket and nematodesmata are not recognizable either *in vivo* or after silver staining. The circumoral kinety consisting of densely spaced dikinetids is situated at the base of the oral bulge, and only one kinetosome of each dikinetid bears a cilium (Figures 5G,J,K and Table 1). There are five perioral kineties, with the anterior two rows composed of dikinetids and the posterior three rows composed of monokinetids (Figures 5G,J,K and Table 1); the perioral cilia are about 9 or 10 μm long (Figure 4C).

### Molecular Phylogeny Based on SSU rRNA Gene Sequences

The SSU rRNA gene sequences of *Lagynus binucleatus* sp. n. and *Foissnerophrys alveolata* gen. n., sp. n. were deposited in the GenBank database with accession numbers, lengths, and guanine–cytosine (GC) content as follows: MW481207, 1,709 bp, 43.42% and MW481206, 1,723 bp, 42.86%, respectively.

**FIGURE 6:**
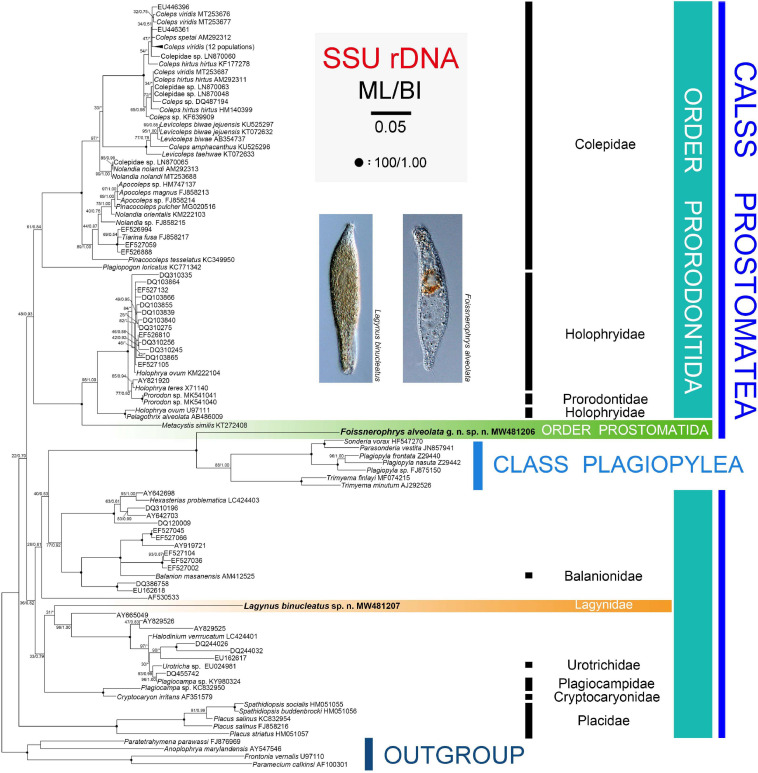
Maximum likelihood (ML) tree inferred from SSU rRNA gene sequences with nodal support for ML and BI analyses. Newly sequenced species, i.e., *Lagynus binucleatus* sp. n. and *Foissnerophrys alveolata* gen. n., sp. n., are in bold. Numbers near nodes denote maximum likelihood (ML) bootstrap values and Bayesian inference (BI) posterior probabilities, respectively. Asterisks (*) reflect disagreements in topology between the BI and ML trees. The scale bar corresponds to 0.05 expected substitutions per site.

The topologies of the SSU rRNA gene trees constructed using ML and BI analyses are similar, therefore, only the ML tree is shown here with support values from both algorithms (Figure 6). Three main subclades are recognizable: (1) Family Placidae forms the basal subclade within the class Prostomatea. (2) Colepidae, Holophryidae, Prorodontidae, and *Metacystis similis* form a subclade with very weak support (ML 48%, BI 0.93). It is noteworthy that *Metacystis similis*, the only available prostomatid sequence, clusters in the clade formed by Prorodontidae and Holophryidae with full support. (3) All other groups, the two new species, and the class Plagiopylea form a subclade with very poor support (ML 36%, BI 0.82). Plagiopyleans nest within the class Prostomatea and cluster with *Foissnerophrys alveolata* sp. n. with maximum support. *Lagynus binucleatus* sp. n., Plagiocampidae, Urotrichidae, and several unidentified environmental sequences form a very weakly supported clade. Therefore, the position of this new species remains unresolved.

### Fluorescence *in situ* Hybridization (FISH) and 16S rRNA Gene Sequence Analysis of Intracellular Prokaryotes

The distributions of bacteria and archaea within the cells of *Lagynus binucleatus* sp. n. were similar, both being concentrated in the anterior half of the body in filamentous or flocculent forms (Figures 2N,O). However, the red fluorescence of the Eub338 probes was relatively stronger in the middle of the body, while conversely, the green fluorescence of the Arc915 probes was stronger in the anterior part of the body. Four bacterial sequences were found with high similarity (> 99.70%) to known sequences, namely, *Corynebacterium tuberculostearicum* (NR028975) (99.86%), *Paracoccus sanguinis* (NR135883) (99.86%), *Leclercia adecarboxylata* (NR104933) (99.80%), and *Brevundimonas vesicularis* (NR037104) (99.71%). Among these, *C. tuberculostearicum* belongs to Actinobacteria, while the rest belong to Proteobacteria, i.e., *P. sanguinis* and *B. vesicularis* (Alphaproteobacteria), and *L. adecarboxylata* (Gammaproteobacteria).

In *Foissnerophrys alveolata* sp. n., the distribution of the two kinds of fluorescence was similar, both being concentrated in the anterior half and the posterior end of the body, but the aggregation shapes differed. Most of the red fluorescence aggregations are spherical or block shaped and appear to be surrounded by food vacuoles (Figure 4K). In contrast, the green fluorescence aggregations are rod shaped or globular and bear a stronger resemblance to archaea (Figure 4L). Furthermore, the green fluorescence is stronger than the red fluorescence in the posterior end of the cell (Figures 4K, L). Four of the bacterial sequences were found with high similarity (>99.70%) to known sequences, namely, *Staphylococcus haemolyticus* (NR113345) (99.93%), *Staphylococcus epidermidis* (NR036904) (99.86%), *Prolinoborus fasciculus* (NR104948) (99.79%), and *Paracoccus sphaerophysae* (NR117441) (99.78%). Two of these (*S. haemolyticus* and *S. epidermidis*) belong to the class Bacilli (phylum Firmicutes), whereas the other two (*Prolinoborus fasciculus* and *Paracoccus sphaerophysae*) belong to the phylum Proteobacteria.

## Discussion

### Morphological Comparison and Systematics of *Lagynus binucleatus* sp. n.

Based on its body shape and infraciliature, especially the brosse and perioral kineties, *Lagynus binucleatus* sp. n. corresponds well with the genus *Lagynus* Quennerstedt, 1867 (Quennerstedt, 1867; Sola et al., 1990; Foissner et al., 1995). Hitherto, only three known congeners have been described, i.e., *L. elegans* (Engelmann, 1862) Quennerstedt, 1867, *L. cucumis* (Penard, 1922) Foissner, 1987, and *L. verrucosa* Foissner, 1983. *Lagynus binucleatus* sp. n. can be easily separated from each of these by having two (vs. one) macronuclear nodules (Figure 2K; Penard, 1922; Foissner, 1983; Sola et al., 1990). In addition, *L. elegans* differs from *L. binucleatus* sp. n. by having three to five conspicuous annular furrows in the neck region (vs. about 11 inconspicuous furrows in the latter) and in the composition of the brosse, i.e., three or four rows, each with four to six kinetosomes in *L. elegans* vs. three rows, each comprising a pair of kinetosomes in *L. binucleatus* sp. n. (Figure 3G; Sola et al., 1990; Foissner et al., 1995) (Table 2).

**TABLE 2:** Comparison of *Lagynus binucleatus* sp. n. with congeners.

Characteristics	*Lagynus binucleatus* sp. n.	*L. elegans* (Engelmann, 1862) Quennerstedt, 1867	*L. cucumis* (Penard, 1922) Foissner, 1987
Body size *in vivo*	165–340 × 20–60 μm	90–200 × 40–60 μm	100–190 μm
Body size after protargol staining	153–310 × 21–79 μm	75.5–116.2 × 40.5–54.6 μm	–
Body shape	Spindle shaped	Bottle shaped	Cylindroid
Presence of distinct neck	No	Yes	No
Ma, number	2	1	1
Ma, shape	Ellipsoidal	Ellipsoidal to reniform	Ellipsoidal
Mi, number	1	1	–
Mi, shape	Ellipsoidal	Ellipsoidal	–
Presence of collecting channels	No	–	Yes
Brosse rows, number	3	3 or 4	–
Number of kinetosomes per brosse row	2	4–6	–
SK, number	28–38	32–50	–
Circumoral kineties, number	1	1	–
Perioral kineties, number	4	3	–
Cervical kineties, number	8–14	3–5	4–5
Data source	Present study	Sola et al., 1990; Foissner et al., 1995	Penard, 1922

*Ma, macronucleus (or macronuclear nodules); Mi, micronucleus; SK, somatic kineties.*

*Lagynus cucumis* is similar to *L. binucleatus* sp. n. in shape (Figures 2A, 3A). Although no infraciliature data are available for *L. cucumis*, these species can be clearly separated by certain characters *in vivo*. For example, *L. cucumis* possesses a shorter body (100–190 μm vs. 180–230 μm), collecting channels that extend from the contractile vacuole to the mid-region of the cell (vs. collecting channels absent in *L. binucleatus* sp. n.), and conspicuous longitudinal grooves on the anterior half of the body (vs. longitudinal grooves absent in *L. binucleatus* sp. n.) (Penard, 1922) (Table 2).

The updated information on the genus *Lagynus* allows a reevaluation of the classification of species *L. verrucosa* Foissner, 1983, and its assignment to the genus *Lagynus*. The perioral kineties of *L. verrucosa* are composed of slightly spiraling longitudinal rows and, hence, very likely correspond to head kineties of the order Lacrymariida Lipscomb and Riordan, 1990. On the other hand, the perioral kineties of *L. elegans* and *L. binucleatus* sp. n. are composed of circular rows (Foissner, 1983). *Lagynus verrucosa* also clearly differs from its congeners in the orientation of its somatic kineties (circularly vs. longitudinal) (Foissner, 1983). Most importantly, no description of *L. verrucosa* has included the brosse, the presence of which is an important diagnostic characteristic of Prorodontida (Corliss, 1974; Lynn, 2008). The presence of spiraling head kineties is a characteristic feature of Lacrymariidae de Fromentel, 1876 (Litostomatea, Lacrymariida) (Quennerstedt, 1867; Kahl, 1930; Foissner, 1983; Foissner et al., 1995). Therefore, we suggest that *L. verrucosa* be transferred to Lacrymariidae, although it cannot be assigned to any known genus within this family (i.e., *Lacrymaria* Bory de Saint Vincent, 1824, *Pelagolacrymaria* Foissner et al., 1999, *Phialina* Bory de Saint Vincent, 1824, and *Phialinides* Foissner, 1988), and may, therefore, represent a new genus (Kahl, 1930; Foissner, 1988; Foissner et al., 1999). Further information on the morphology and molecular phylogeny of *L. verrucosa* is needed in order to determine its correct systematic position.

In the SSU rRNA gene tree, *Lagynus binucleatus* sp. n. branches independently from other families of the order Prorodontida, thus, supporting the validity of the family Lagynidae. Furthermore, Lagynidae is more closely related to Cryptocaryonidae, Plagiocampidae, and Urotrichidae, than to other families of the order Prorodontida. However, these findings are based on very limited taxon sampling and on only a single gene. Therefore, more data are needed in order to determine the evolutionary relationships within the Prorodontida.

### Morphological Comparison and Systematics of *Foissnerophrys alveolata* gen. n., sp. n.

Based on the apical cytostome and the lack of brosse and toxicysts, *Foissnerophrys alveolata* sp. n., should be assigned to the order Prostomatida, which comprises only two families, i.e., Apsiktratidae Foissner et al., 1994, and Metacystidae Kahl, 1926 (Lynn, 2008). The new taxon can be distinguished from the Apsiktratidae by having a spindle-shaped (vs. ovoidal) body and perioral kineties (vs. lacking in the latter), and by the absence (vs. presence) of a collar-like appendix at the anterior end of the body (Foissner et al., 1994). In having an alveolar layer over the cortex and conspicuous circumferential paratenes (perioral kineties) around the neck region, *F. alveolata* gen. n., sp. n. resembles several species of Metacystidae (Figures 4A,I, 5G,H) (Kahl, 1930). The Metacystidae currently include three genera, but the new species cannot be assigned to any of them.

*Metacystis* Cohn, 1866, can be separated from *Foissnerophrys alveolata* sp. n. by the presence (vs. absence) of a terminal vacuole, the presence (vs. absence) of a caudal cilium, the orientation of the somatic kineties (transverse vs. longitudinal), and the cylindrical (vs. spindle-shaped) body (Kahl, 1930; Small and Lynn, 1985) (Table 3).

**TABLE 3:** Comparison of *Foissnerophrys* gen. n. with related genera.

Genus	*Foissnerophrys* gen. n.	*Metacystis* Cohn, 1866	*Vasicola* Tatem, 1869	*Pelatractus* Kahl, 1930
Body shape	Spindle shaped	Cylindrical	Ovoid	Spindle shaped
Body tapered at rear	Yes	No	Yes	Yes
SK, orientation	Longitudinal	Transverse	Transverse	Longitudinal
Presence of monokinetid oral ring	No	No	Yes	No
Presence of terminal vacuole	No	Yes	No	Yes
Presence of bulge on terminal vacuole	–	Yes	–	No
Presence of caudal cilium	No	Yes	Yes	No
Caudal cilium, number	–	One	One or more	–
Presence of lorica	No	Yes or no	Yes	Yes
Presence of CV	No	Yes or no	Yes or no	Yes
CV, position	–	Mid–body	Terminal	Mid–body
CV, number	–	One	One	One or more
Data source	Present study	Kahl, 1930; Small and Lynn, 1985	Kahl, 1930; Small and Lynn, 1985	Kahl, 1930; Small and Lynn, 1985

*CV, contractile vacuole; SK, somatic kineties.*

*Vasicola* Tatem, 1869, clearly differs from *Foissnerophrys alveolata* sp. n. in body shape (ovoidal vs. spindle-shaped), the orientation of the somatic kineties (transverse vs. longitudinal), and the presence (vs. absence) of a lorica (Kahl, 1930) (Table 3).

*Pelatractus* Kahl, 1930 most closely resembles *Foissnerophrys alveolata* sp. n. in terms of its spindle-shaped body, longitudinal somatic kineties, and the absence of a caudal cilium. Nevertheless, the former can be distinguished from *F. alveolata* sp. n. by the presence (vs. absence) of a lorica, the presence (vs. absence) of a terminal vacuole, and by the presence (vs. absence) of one or more contractile vacuoles (Kahl, 1930; Small and Lynn, 1985) (Table 3).

In the SSU rRNA gene tree, *Foissnerophrys alveolata* gen. n., sp. n. was closely related to the class Plagiopylea. However, *Metacystis similis* KT272408 failed to cluster with the new species and instead was sister to the Prorodontidae + Holophryidae clade with full support. Therefore, the findings of the present and previous studies indicate that the brosse might not be a suitable character for distinguishing between the orders Prostomatida and Prorodontida. Consequently, more work needs to be carried out to answer this question and to determine the evolutionary relationships within the class Prostomatea.

In conclusion, both the morphological and the molecular data indicate that our new species cannot be assigned to any extant genus. A new prostomatid genus, *Foissnerophrys* gen. n., is, thus, proposed. As mentioned above, familial assignment of the new genus cannot be determined at present. *Foissnerophrys* gen. n. should, therefore, be classified as incertae sedis within the order Prostomatida.

### The Distribution and Identification of Intracellular Prokaryotes

With the development of molecular techniques, an increasing number of endosymbiotic prokaryotes have been discovered in anaerobic ciliates. Most archaeal symbionts in these ciliates are methanogens (Fenchel and Finlay, 1991; Embley and Finlay, 1993; Fenchel, 1993; Finlay et al., 2000; Shinzato et al., 2007; Hackstein et al., 2008), and most bacterial symbionts are from the phylum Proteobacteria (Fokin et al., 2000; Beier et al., 2002; Edgcomb et al., 2011; Schrallhammer et al., 2011; Boscaro et al., 2012; Vannini et al., 2012; Gong et al., 2014). In contrast, bacterial symbionts of other phyla, such as Firmicutes and Verrucomicrobia, seem to be rare (Petroni et al., 2000; Shinzato et al., 2007). Prostomateans, especially in the genera *Coleps* Nitzsch, 1827, *Holophrya* Ehrenberg, 1831, and *Urotricha* Claparède and Lachmann, 1859, have been reported to feed on a variety of bacteria (Madoni et al., 1990; Epstein and Shiaris, 1992; Šimek et al., 1996). Furthermore, symbiotic bacteria have been reported in *Holophrya* sp., *Holophray teres* (Ehrenberg, 1834) Foissner et al., 1994, and *Urotricha ovata* Kahl, 1926 (de Puytorac and Grain, 1972; Fokin, 2012). Although we did not obtain sequences of archaea, sequences of bacteria belonging to the phylum Proteobacteria were detected in both *L. binucleatus* sp. n. and *F. alveolata* gen. n., sp. n. In the absence of starvation culture and environmental sequencing, we cannot be sure whether these prokaryotes were ingested food organisms or endosymbionts. However, we can be sure that the bacteria associated with prostomateans are closely related to species commonly found in planktonic and benthic habitats, i.e., according to our 16S rRNA data, they had 99.70% similarity to sequences of *Brevundimonas vesicularis*, *Leclercia adecarboxylata*, *Paracoccus sanguinis*, *Paracoccus sphaerophysae*, and *Prolinoborus fasciculus*. We believe that these initial attempts are meaningful for research on microbial food webs and symbiotic relationships between eukaryotes and prokaryotes.

## Data Availability Statement

The datasets presented in this study can be found in online repositories. The names of the repository/repositories and accession number(s) can be found below: https://www.ncbi.nlm.nih.gov/, MW481207, https://www.ncbi.nlm.nih.gov/, MW481206, https://www.ncbi.nlm.nih.gov/, MW979568, https://www.ncbi.nlm.nih.gov/, MW979569, https://www.ncbi.nlm.nih.gov/, MW979570, https://www.ncbi.nlm.nih.gov/, MW979571, https://www.ncbi.nlm.nih.gov/, MW979572, https://www.ncbi.nlm.nih.gov/, MW979573, https://www.ncbi.nlm.nih.gov/, MW979574, and https://www.ncbi.nlm.nih.gov/, MW979575.

## Author Contributions

XH conceived and designed the manuscript. LJ carried out the live observation, protargol staining, and wrote the manuscript. WZ performed the data analyses. HE-S, SA-F, and AW revised the writing of the manuscript. All authors contributed to the article and approved the submitted version.

## Conflict of Interest

The authors declare that the research was conducted in the absence of any commercial or financial relationships that could be construed as a potential conflict of interest.
